# Occurrence and Distribution of bovine tuberculosis (*Mycobacterium bovis*) in Slaughtered cattle in the abattoirs of Bauchi State, Nigeria

**DOI:** 10.14202/vetworld.2015.432-437

**Published:** 2015-03-30

**Authors:** Adamu Saleh Saidu, E. C. Okolocha, A. A. Gamawa, M. Babashani, N. A. Bakari

**Affiliations:** 1 Department of Veterinary Public Health and Preventive Medicine, Ahmadu Bello University, PMB, 1013, Zaria, Kaduna State, Nigeria; 2 Department of Animal Health, College of Agriculture, Bauchi State, Nigeria; 3 Veterinary Teaching Hospital, Ahmadu Bello University, PMB, 1013, Zaria, Kaduna State, Nigeria

**Keywords:** abattoir, Bauchi State, bovine TB, PCR, post-Mortem, Ziehl-Neelsen

## Abstract

**Aim:**

This study was aimed to determine the prevalence of bovine tuberculosis (bTB) in slaughtered cattle in Bauchi State, Nigeria. The cause (s) of grossly suspected bTB lesions encountered at the abattoirs during post-mortem (PM), as whether due to *Mycobacterium bovis* alone or together with other acid fast bacilli (AFB).

**Materials and Methods:**

A cross-sectional abattoir based study was conducted on 800 cattle slaughtered in the Northern, Central and Southern zonal abattoirs of Bauchi State, Nigeria, from June to August 2013; using PM meat inspection, Ziehl-Neelsen staining (ZN) and confirmatory polymerase chain reaction (PCR) techniques.

**Results:**

The occurrence of bTB lesions from the organs of slaughtered cattle in Bauchi State, showed that the lungs had the highest number of suspected tissues 65 (54.20%), followed by the lymph nodes 28 (23.30%) while the heart, liver, spleen, intestines and mammary glands had the other 8.3%, 6.7%, 5.0%, 1.7%, and 0.8%, suspected tissues respectively. By ZN microscopic staining all 100% (2/2) of the intestines were positive for bTB, followed by the heart with 50% (5/10), then the lungs 29.23% (19/65); while the liver, lymph nodes, and spleen had 25%, 21.43% and 16.67% respectively were tested positive for bTB. It was only the mammary gland that tested negative for bTB in all the suspected tissues sampled. By PCR, the intestines had the highest positive bTB with 100% (2/2), followed by the liver with 12.5% (1/8), and then the lungs with 7.8% (5/65). The lymph nodes had 7.14% (2/28) tissues that tested positive for bTB. However, the spleen, heart and mammary gland were all tested negative with 0%; indicating that the false positive for bTB detected by ZN were confirmed by PCR. While based on the location of the abattoirs in the three senatorial zones of Bauchi State, Bauchi zonal abattoir had the highest number of suspected bTB cases 75 (62.50%), followed by Katagum zonal slaughter house with 32 (26.7%) and then Misau with 13 (10.8%). By the ZN staining technique, there were 25 (33.33%) positivity in Bauchi Zonal abattoir, while Katagum and Misau abattoirs had 9 (28.13%) and 1 (7.72%) positive respectively. By the PCR technique, 9 (12.00%), 1 (3.13%) and 0 (0.00%) positive cases were recorded for Bauchi, Katagum and Misau abattoirs respectively.

**Conclusion:**

The present study estimated the prevalence rate of bTB in Bauchi State, using PM, ZN and PCR techniques at 15.0%, 29.16% and 8.33%, respectively. Bovine TB lesions found at PM were not all due to *M. bovis* alone, as other MTBC and AFB organisms may cause bTB-like lesions that were excluded by PCR specific primers. The prevalence of bTB was higher in Bauchi abattoir that supplies larger population of the state with beef. These findings also demonstrate the urgent need for public health authorities in the state to intervene in the control of the zoonotic bTB.

## Introduction

Tuberculosis (TB) remains a major global public health problem. The World Health Organization (WHO) estimates that there are 8 million new cases and 3 million deaths directly attributed to the disease each year [1]. This makes TB the leading cause of death in many resource-poor and developing countries. TB is an important disease in humans and animals worldwide. The tubercle bacillus infects over 2 billion people or one-third of the world’s population and it is estimated that 1.5-2 million people die from TB each year [2]. Significant progress has been made toward the elimination of TB caused by *Mycobacterium* TB complex (MTBC) from humans in industrialized countries [3]. However, in many countries where financial resources are insufficient to support TB programs, only limited progress has been made toward the control of the disease. The development of multi-drug resistant and extensively drug-resistant (MDR and XDR-TB) TB strains have contributed to difficulty, the development of efficacious regimens for the treatment of TB in humans and has significantly increased the cost associated with the use of multiple drug therapies [4].

Bovine TB (bTB) is a chronic infectious and contagious zoonotic disease of domestic animals, wild animals and humans [5]. It also occurs in a wide range of mammalian species [6]. It is characterized by the formation of granulomas in tissues, especially in the lungs, lymph nodes, liver, intestines and kidney [7,8]. In Nigeria, there have been limited studies to determine the prevalence or relationship between bovine and human TB, especially with the emerging culture of eating improperly cooked beef and mutton, along with the drinking of unpasteurized fresh milk [9,10]. Furthermore, Cadmus and Adesokan (2009) reported an economic loss of N13, 871,014/annum with associated public health implications due to TB (7.95%) as major reasons for condemnations in some abattoirs in Western-Nigeria. The majority of these occur in the developing nations [11].

Bovine TB is caused by *Mycobacterium bovis* that is a member of MTBC [12,13]. The etiological agents of mammalian TB, classified as members of the *Mycobacterium* TB complex (MTBC), include: *Mycobacterium* TB, *Mycobacterium bovis, Mycobacterium microti, Mycobacterium caprae, Mycobacterium africanum, Mycobacterium canettii*, and *Mycobacterium pinnipedii*. *Mycobacterium africanum* consists of a rather heterogeneous group of strains isolated from humans in Africa [14]*. Mycobacterium bovis*, otherwise known as the bovine tubercle bacillus is the cause of bTB, and the organism may be transmitted by aerosol or droplets of exudates containing the bacilli. It can be transmitted by ingestion of feed and water contaminated with urine, fecal material or exudates that contain the tubercle bacilli from diseased animals [15]. The bovine tubercle bacilli is usually assigned to bTB in cattle, but still is often used to denote bovine strain of the tubercle bacillus irrespective of the host. The bovine tubercle bacillus has one of the broadest host ranges of all known pathogens. The species has been reported in domesticated and feral bovidae. Other animal species in which the disease has been reported include goat, sheep, pig, horse, cat, dog, fennac fox, bison, buffalo, badger, wild and feral pig, antelope, camel, man and non-human primates, among others [16]. Cattle movement, particularly from areas where bTB is reported, is the best predictor of disease occurrence [17].

In rare cases, humans can become infected with *M. bovis* via direct inoculation (Grange, 2001). Referred to as the Butcher’s Wart (analogous to the Prosector’s Wart, which is caused by *M*. TB and is an occupational risk associated with performing autopsies), this skin lesion can occur in persons handling infected meat. It is very rare and generally self-limiting. Because *M. bovis* is either enzootic or found sporadically in much of the developing world, there is clearly a risk of cow to human transmission by either ingestion or inhalation [18].

As a result of the lack of surveillance data, the actual scope of the problem is unknown. However, our abattoir settings in Nigeria could always be a hazard and critical control points where thorough examination are needed to control the entry of foodborne pathogens (*M. bovis*) to retail markets and finally to the consumers (public).

## Materials and Methods

### Ethical approval

The abattoirs included in the research are all legal and government approved.

### Study design

Bauchi State occupies a total land area of 49,119 Km² representing about 5.3% of Nigeria’s total land mass [19]. The State is bordered by Kano and Jigawa to the North, Taraba and Plateau to the south, Gombe and Yobe to the East and Kaduna to the West. The State is highly populated with cattle mainly owned by Fulani herdsmen. The cattle population is estimated at 1,789,000 out of the Nigerian cattle population of 13,900,000 [20]. Bauchi State has a total of 55 tribal groups in which Hausa, Fulani, and Kanuri, are the main tribes. The study was carried out in Bauchi, Katagum and Misau Local Government Areas abattoirs (out of the 20 LGAs) of Bauchi State, each representing a senatorial zone namely Bauchi South, Bauchi North and Bauchi Central respectively [21].

A 3-month cross-sectional study was carried out in the Northern, Central and Southern Senatorial zones of Bauchi State, Nigeria. Post-mortem (PM) examination of carcasses, as a diagnostic technique, Ziehl-Neelsen (Z-N) staining and polymerase chain reaction (PCR) were used to determine the prevalence of bTB in some Local Government Areas in the State while questionnaire administration was used to determine the public health awareness of the people at risk (butchers, veterinarians, and other abattoir personnel) were carried out. The three major slaughter houses namely Bauchi, Misau, and Katagum (Azare) in each of the three senatorial zones of Bauchi State were selected for the study for sampling purposes, based on their daily slaughter capacity and socio-economic impact of the abattoir. Accordingly, out of the 800 cattle examined (sample size), a proportionate distribution was employed, given a share of 50% (400) to Bauchi abattoir and 25% (200) each to Katagum and Misau slaughter houses. This was based on their daily slaughter capacity of 40-60, 20-30 and 20-25 cattle from Bauchi abattoir, Katagum, and Misau slaughter houses, respectively.

### Study animals

Cattle slaughtered at Bauchi, Katagum and Misau abattoirs were the subjects of the study. Their respective breeds, sexes, and ages were recorded during sampling.

### Abattoir inclusion criteria

Abattoir selection was based on convenient and the daily slaughter capacity of the abattoirs, accessibility of the abattoir to the public and its socio-economic impact on the State. The Bauchi abattoir along with Katagum and Misau abattoirs were therefore selected.

### Sampling

#### Sample size determination

N=Z^2^ PQ/d^2^ - Ass described by [22]

Where: N = sample size

Z = value for the corresponding confidence level (e.g., 1.96 for 95% confidence)

P = estimated value for the proportion of a sample/expected prevalence

Q= 1-P, d= is the margin of error (e.g., 1= ± 10%)


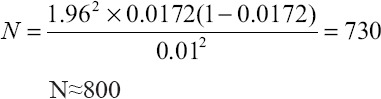


A sample size of 730 cattle was estimated from the expected prevalence rate of 1.72% [23] as described by [22]. However, 800 slaughtered cattle were examined for bTB like-lesions from the selected zonal abattoirs of Bauchi State, for proportionate sampling. The sources of animals for slaughter at the three abattoirs under the study were retrieved from the butchers at the various locations.

### Sampling procedure and transportation

Cattle brought in for slaughter were routinely subjected to procedures such as ante-mortem inspection and PM examination (by meat inspectors), before the carcasses were conveyed to the market. PM examinations of the carcasses were carried out by carefully inspecting the lymph nodes of the head, tonsils, thoracic cavity, and abdomen, including deep and superficial cervical, popliteal and sacral lymph nodes. The meat inspection procedures employed visual examination and palpation of the lungs, liver, kidneys, lymph nodes of the carcass including the mesenteric lymph nodes and intestines. For this study, diagnosis of bovine TB was based on gross detection of typical tubercle, yellowish granulomatous caseated lesions or sometimes ‘gritty’ calcification in carcasses in the above-mentioned organs on incision and with the use of protective clothing. In addition, all nodular lungs were carefully inspected visually, palpated and incised [24-26]. Aging of cattle was carried out in the abattoir after slaughter as described by [27]; using the time of appearance of and the degree of wear on the temporary and permanent teeth, taking note of their sex and breeds. *M. bovis* diagnosis was confirmed following proper meat inspection by microbiological identification and characterization of the etiological agent using ZN stain and PCR, respectively.

All suspected tissue samples from cattle with suspected bTB-lesions were collected into sterile screw capped containers (with 9% normal saline solution to keep them moist) and transported inside a cooler containing ice pack to the Department Veterinary Public Health and Preventive Medicine, Bacterial Zoonoses Laboratory, Ahmadu Bello University, Zaria and stored at −20°C until analyzed. Stored samples were processed for microscopic examination (Modified ZN staining technique).

### Microscopic examination

#### Acid fast/ZN staining technique

All suspected tissues that were collected from the abattoirs and stored at −20°C were thawed to room temperature and then processed for ZN Staining technique according to the method of [28], to detect acid - fast bacilli. The results were interpreted by the presence of red, straight or slightly curved rod occurring singly or in a cluster indicated the presence of tubercle bacilli (Acid Fast Bacilli [AFB] positive (Plate II).

### Molecular detection of *M. bovis* from tissues

The tissue samples positive by ZN were collected into a 1.5 ml microfuge tube containing lysis buffer, stored at room temperature and transported on ice pack to DNA-Labs, Kaduna. About 1 g of the tissue sample was homogenized with pestle and mortar for chromosomal DNA extraction using a phenol-chloroform technique as described by [29,30]. The supernatant was discarded; the suspension was then cooled at 4°C, neutralized with 3 volumes of 0.1 m Tris-HCL (PH. 7.4) buffer, and centrifuged (5,000 × g, 5 min) to get rid of the tissues’ membrane and possible contaminants. The pellet was dried and re-dissolved in 20 μl of 1 × TE buffer and DNA was precipitated with ethanol, collected by centrifugation and dissolved in 50 μl of distilled water. 5 μl of the extracted DNA was run on 1.0% agarose gel and spectrophotometer to confirm the presence of DNA. The remaining DNA samples were stored at −20°C until further use.

### PCR amplification procedures

A volume of 10 µl suspended DNA was used as a template for PCR amplification under standard conditions as described by [31]. A commercial “Hot-Stat” PCR Premix (Bioneer, USA), a mixture prepared in a lyophilized format containing: Taq DNA polymerase, Reaction buffer, dNTPs (dATP, dGTP, dCTP, dTTP) and MgCl_2_ was used. All amplification reactions were performed using a Perkin Elmer Thermocycler (Perkin Elmer Cetus) programed for 40 amplification cycles. The reaction was performed in a final volume of 50 μl containing 10 μl of DNA Template, ×1 TE reaction buffer (containing 10 mM Tris-HCl (pH 8.3), 50 mM KCl, 1.3mM MgCl_2_, and 0.001% of gelatin), 2.5 U Taq polymerase, 0.2 mM of each deoxynucleoside triphosphate, and 75 pmol of each primer (forward and reverse primers; 5’CCCGCTGATGCAAGTGCC3’ and 5’CCCGCACATCCCAACACC3’). DNA from *M. bovis* ATCC 19210 and BCG Pasteur 27291 and sterile nuclease-free water were used as positive and negative PCR controls, respectively. After an initial denaturation step (at 94°C, for 5 min.), 40 amplification cycles were performed as follows: Denaturation at 94°C for 1 min, annealing at 58°C for 30 s and extension at 72°C for 30 s; with an increment of 1 s per cycle for the denaturation and extension segment. A final extension was performed at e72°C for 15 min [31].

## Results

**Figure-1:**
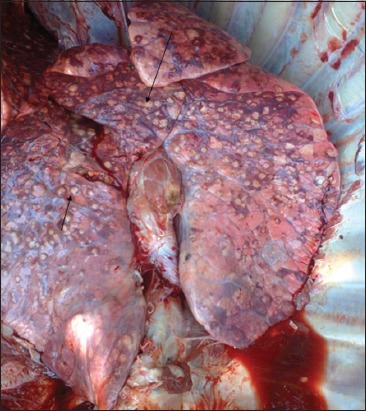
Photograph of the affected lungs showing massive granulomatous (tuberculous) lesions from slaughtered cattle, during Post-Mortem. Legend: See the arrows pointing at the tuberculous lesion].

The occurrence of bTB lesions from the organs of slaughtered cattle in Bauchi State, showed that the lungs had the highest number of suspected tissues 65 (54.20%), followed by the lymph nodes 28 (23.30%) while the heart, liver, spleen, intestines and mammary glands had the other 8.3%, 6.7%, 5.0%, 1.7%, and 0.8%, suspected tissues respectively (Table-1). By ZN microscopic staining all 100% (2/2) of the intestines were positive for bTB, followed by the heart with 50% (5/10), then the lungs 29.23% (19/65); while the liver, lymph nodes, and spleen had 25%, 21.43% and 16.67% respectively that tested positive for bTB. It was only the mammary gland that tested negative for bTB in all the suspected tissues sampled. By PCR, the intestines had the highest positive bTB with 100% (2/2), followed by the liver with 12.5% (1/8), and then the lungs with 7.8% (5/65). The lymph nodes had 7.14% (2/28) that tested positive for bTB. However, the spleen, heart and mammary gland were all tested negative with 0% (0/6), 0% (0/10) and 0% (0/1) respectively; (Table 1). Figure-1 presents a typical lesion of bTB encountered at the abattoir during PM of the study.

**Table-1:** Distribution and occurrence of suspected bTB lesions in slaughtered cattle in Bauchi State, Nigeria.

Organs examined	No. TB suspected lesions (%)	ZN staining (%+ve)	PCR (%+ve)
Lungs	65 (54.2)	19 (29.23)	5 (7.80)
Lymph nodes	28 (23.3)	6 (21.43)	2 (7.14)
Intestines	2 (1.7)	2 (100)	2 (100)
Liver	8 (6.7)	2 (25)	1 (12.50)
Spleen	6 (5.0)	1 (16.67)	0 (0)
Heart	10 (8.3)	5 (50)	0 (0)
Mammary gland	1 (0.8)	0 (0)	0 (0)
Total	120	35	10

**Figure-2:**
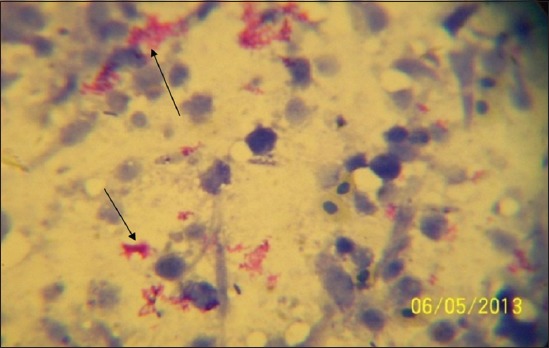
Micrograph showing the Acid Fast Bacilli (AFB) organisms under a microscope (×100 - oil immersion). Legend: See arrows pointing at the red coloured AFB cluster on the methylene blue background after the Ziehl-Neelsen (ZN) staining.

In Table 2, based on the location of the abattoirs in the three senatorial zones of Bauchi State, Bauchi zonal abattoir had the highest number of suspected bTB cases 75 (62.50%), followed by Katagum zonal slaughter house with 32 (26.7%), and Misau 13 (10.8%). By the ZN staining technique, there were 25 (33.33%) positivity in Bauchi Zonal abattoir, with Katagum and Misau abattoirs having 9 (28.13%) and 1 (7.72%) positives respectively. With regards to the PCR technique, 9 (12.00%), 1 (3.13%) and 0 (0.00%) positive cases were recorded for Bauchi, Katagum and Misau abattoirs respectively (Table 2). Figure-2 presents a typical micrograph of AFB (*M. bovis* and other MTBC) identified from suspected tissue sample after the Ziehl-Neelsen (ZN) staining.

**Table-2:** Distribution of suspected bTB in the three selected LGA abattoirs of Bauchi State, Nigeria.

Zones	No. sampled (%)	ZN Stain (%+ve)	PCR (%+ve)
Bauchi (South)	75(62.5)	25 (33.33)	9 (12.00)
Katagum (North)	32(26.7)	9 (28.13)	1 (3.13)
Misau (Central)	13(10.8)	1 (7.7)	0 (0)
Total	120	35	10

## Discussion

The findings that, 120 (15%) bTB suspected lesions were observed in the 800 slaughtered cattle examined in Bauchi State abattoirs had emphasized the importance of PM meat inspection. This agrees with the report of [32], as PM examination remains the immediate diagnostic tool to be used in endemic slaughter houses. The distribution and occurrence pattern of suspected bTB lesions in different organs of slaughtered cattle in Bauchi State, agreed with the retrospective study reported by Igbokwe et al [33] on gross bTB lesions in different organs in slaughtered cattle in Maiduguri, Nigeria.

The present study also showed that, the highest number of suspected bTB cases was recorded in Bauchi abattoir, followed by Katagum, and then Misau with the least number. This could be due to the socio-economic impact, as a result of high patronage with the resultant increase in daily slaughter capacity associated with the Bauchi metropolitan abattoir than the other two abattoirs. Many researchers have reported relatively higher prevalence rates of bTB in various parts of Africa. In Chad, about 9% of the inspected cattle were condemned because of bTB in slaughter houses as reported by Maho et al [34]. Ibrahim et al [35] reported a prevalence rate of 13.2% of bTB in Tanzania using a tuberculin testing. All these differences were due to the different techniques used in their studies. Thus, the differences in prevalence rates may be due to possible false positive results by their procedures, and perhaps differences in socio-geographic pattern of cattle distribution could also be a factor [36]. A higher prevalence rate recorded in this study as compared to earlier reports of 1.44% and 2.80% also based on abattoir records by [37,38] in Western-Nigeria and Ethiopia respectively. However, A little later thereafter by [11], a relatively similar prevalence rate of 7.95% was reported in an abattoir based study in Western-Nigeria. Clearly indicating that bTB is still endemic in many African countries. The present study found that suspected bTB lesions encountered in slaughtered cattle at PM may not be caused by *M. bovis* alone as MTBC and other AFB organisms, like *Norcardia asteroides* may be involved while 35 (29.16%) of the 120 suspected bTB lesions were positive by ZN.

The prevalence rates shown in this study is much higher in female cattle than in male cattle. However, there was no statistically significant association between sex of cattle with bTB by ZN technique as earlier observed by [10] and also using the PCR technique. This was also in agreement with the expected sex-distribution pattern of the disease in cattle as reported by [39]; so also by [40] and [41] who reported the same findings. Because female cattle stay longer in the herds than male ones and the fact that they are somewhat immunocompromised during pregnancy and lactation that may lead to conversion of latent TB infection to an active TB disease could be responsible for the disparity recorded in this study. A similar finding was previously reported by [32], in which a review of bTB in Nigeria showed there was no statistically significant association between sex of cattle with bTB by ZN. However, on the contrary, another abattoir based study on slaughtered camels was conducted in Kano, North Western Nigeria by [40] using ZN, in which a relatively lower prevalence rate of 16.60% was obtained. They also found a higher prevalence rate in male than in female camels. This may be attributed to different animal species used in the study.

The age-related increase in the prevalence rate shown in this study by both ZN and PCR was consistent with the chronic pattern of TB in cattle reported by [39,40]. Animals are increasingly becoming exposed in endemic situations where the infection is rampant and showing increasing positivity with age [38]. The statistically significant higher prevalence rate associated with the age found in this study might have explained the chronic nature of the disease in cattle and it is consistent with the finding of high infection rates with increasing age of animal as reported much earlier by [10] and much later by [41]. In addition, in stress and old age situations, a latent infection may be reactivated and lead to the development of an active disease, as reported by [42].

## Conclusion

The present study estimated the prevalence rate of bTB in Bauchi State, using PM, ZN and PCR techniques at (15.0%, 29.16% and 8.33%, respectively). Bovine TB lesions found at PM were not all due to *M. bovis* alone, as other MTBC and AFB organisms may cause bTB-like lesions that were excluded by PCR specific primers. The study recommended that, a proper PM meat inspection should be practiced efficiently at the abattoirs and slaughter houses, before taking beef to the retail markets and thereby to the public. The emergence MDR and XDR-TB strains are also a major concern in Nigeria. Thus, further molecular epidemiological studies with more improved techniques, like MIRU-VNTR and Spoligotyping, should be carried out on isolates from the state to look for other zoonotics, like *M. africanum*.

## Authors’ Contributions

ASS: Designed the study, did the sampling, run the laboratory techniques including the Ziehl-Neelsen staining technique and wrote the first draft of the manuscript. ECO: Supervised the whole research and reviewed the manuscript. AAG and MB: Contributed in the data collation and managed the data analyses of the study. NAB: Did the literature searches, part of data analyses and financial contribution. All authors read and approved the final manuscript.
